# Supplementary material for: Rapid turnover of life-cycle-related genes in the brown algae
Source: Genome Biol. 2019 Feb 14;20:35. doi: 10.1186/s13059-019-1630-6 (PMC6374913; doi:10.1186/s13059-019-1630-6)
Supplement: Supplementary file 3 — Protein alignments, corresponding Gblocks html file and CDS sequences for the lineage conserved gametophyte-biased genes. (ZIP 2109 kb) [file 13059_2019_1630_MOESM3_ESM.zip › Con_GA_alignments/Ec-18_000710.aa_ali.fasta-gb.htm]

Ec-18\_000710.aa.fasta.trans.aa\_ali.fasta


## Gblocks 0.91b Results

Processed file: **Ec-18\_000710.aa.fasta.trans.aa\_ali.fasta**  
Number of sequences: **2**  
Alignment assumed to be: **Protein**  
New number of positions: **348** (selected positions are underlined in blue)

```
                         10        20        30        40        50        60
                 =========+=========+=========+=========+=========+=========+
Ec-18_000710     MSARSADTARERGRTVTALLAASATT-PSNPWSRSHKSRSFNDNSNNIR----GGSYAVG
MSTRG.53501.1    MSARSADTARERGRAVDALIAASATELPSTPWSRDHESGKLNVTDGRARRCNGDGPLSPH
                 #########################  ######################           


                         70        80        90       100       110       120
                 =========+=========+=========+=========+=========+=========+
Ec-18_000710     YRRPTTTDPAADDQT--TTTDCGETTLGDGDDDLVRVVDRLLSSKG---QDEGDSLDLCS
MSTRG.53501.1    NDRRSCDHRSSETAADKSRSDDGTMTLNGGDDDLVGAVDFLLSSGGGRTRDPEGDLDLCS
                                     ##########################    ##########


                        130       140       150       160       170       180
                 =========+=========+=========+=========+=========+=========+
Ec-18_000710     STWQSVETMARALATGQSVVSTISRCYRLPGLGRNNSGPQVAEAVRCKARRAGTEAMHSL
MSTRG.53501.1    STWKSVETMARALAAGQSVLTTISRCYSLPDLARNSSPPDAAADLERSAREAGTEMMHSS
                 ############################################################


                        190       200       210       220       230       240
                 =========+=========+=========+=========+=========+=========+
Ec-18_000710     LLSGRFAAKALAIFRRLGQAPLTHPDSFFINSSKLPEAAKALDPGLDDATIRAWMKANGF
MSTRG.53501.1    LISGRFAAHALAIFRRLGQAPPAQPDSLYLNSSRLPEAVRTLDPGVDDATIRAWMKANGF
                 ############################################################


                        250       260       270       280       290       300
                 =========+=========+=========+=========+=========+=========+
Ec-18_000710     EQQARPQPDSLEALSWTEYRQALSELCPAFRRHGISPTTSTERNTAVSTPIDTKHIGNGG
MSTRG.53501.1    ENQDKS-TESLEALSWTEYRKALSELSPAFRRHGISPETSRERKIATTTPVGTIRLARGS
                 ###      ###################################################


                        310       320       330       340       350       360
                 =========+=========+=========+=========+=========+=========+
Ec-18_000710     GS-GGLALSASCPSF-TDHDP---WEAAVDRTPACPSPARPRRRAGLEDDGDTHAVGGRL
MSTRG.53501.1    GCHADISSSRNIPTVRANHSPRGHSEDVVDRTASCTSPLWQHSTVS-ESGGRIRPLENEM
                 #                        #############                      


                        370       380       390       400       410       420
                 =========+=========+=========+=========+=========+=========+
Ec-18_000710     GNCPPRSPGADSYGVLRRKRHAAVSLDYEAITRERPQVMPPLDPSILVDHIKAQGRRDRK
MSTRG.53501.1    GDNPPRSPGADSYGVLRRKRHAAVSVDYEAITKQLPQVMQPLDPSILVDHIKTQGRRERK
                 ############################################################


                        430       440       450       460       470       480
                 =========+=========+=========+=========+=========+=========+
Ec-18_000710     NPGGEEGGRSSLWTLSTHERLLKIPLEKEQVLRCSGRRLLLRIGFDIDGGGLFSTPHFLA
MSTRG.53501.1    NPGSE-GGGVSLWSLDPH------------------------------------------
                 ##### ############                                          


                        490
                 =========+=========
Ec-18_000710     ERARRFDNATALCVVRQST
MSTRG.53501.1    -------------------
```

```
Parameters used
Minimum Number Of Sequences For A Conserved Position: 2
Minimum Number Of Sequences For A Flanking Position: 2
Maximum Number Of Contiguous Nonconserved Positions: 8
Minimum Length Of A Block: 10
Allowed Gap Positions: None
Use Similarity Matrices: Yes
```

```
Flank positions of the 8 selected block(s)
Flanks: [1  25]  [28  49]  [81  106]  [111  243]  [250  301]  [326  338]  [361  425]  [427  438]  

New number of positions in Ec-18_000710.aa.fasta.trans.aa_ali.fasta-gb:  348  (69% of the original 499 positions)
```
